# The Service User Technology Acceptability Questionnaire: Psychometric Evaluation of the Norwegian Version

**DOI:** 10.2196/10255

**Published:** 2018-12-21

**Authors:** Astrid Torbjørnsen, Milada C Småstuen, Anne Karen Jenum, Eirik Årsand, Lis Ribu

**Affiliations:** 1 Department of Nursing and Health Promotion Faculty of Health Sciences OsloMet – Oslo Metropolitan University Oslo Norway; 2 General Practice Research Unit Department of General Practice, Institute of Health and Society University of Oslo Oslo Norway; 3 Norwegian Centre for E-health Research University Hospital of North Norway Tromsø Norway; 4 Department of Clinical Medicine Faculty of Health Sciences UiT The Arctic University of Norway Tromsø Norway

**Keywords:** acceptability, factor analysis, health care, mHealth, telemedicine

## Abstract

**Background:**

When developing a mobile health app, users’ perception of the technology should preferably be evaluated. However, few standardized and validated questionnaires measuring acceptability are available.

**Objective:**

The aim of this study was to assess the validity of the Norwegian version of the Service User Technology Acceptability Questionnaire (SUTAQ).

**Methods:**

Persons with type 2 diabetes randomized to the intervention groups of the RENEWING HEALTH study used a diabetes diary app. At the one-year follow-up, participants in the intervention groups (n=75) completed the self-reported instrument SUTAQ to measure the acceptability of the equipment. We conducted confirmatory factor analysis for evaluating the fit of the original five-factor structure of the SUTAQ.

**Results:**

We confirmed only 2 of the original 5 factors of the SUTAQ, *perceived benefit* and *care personnel concerns*.

**Conclusions:**

The original five-factor structure of the SUTAQ was not confirmed in the Norwegian study, indicating that more research is needed to tailor the questionnaire to better reflect the Norwegian setting. However, a small sample size prevented us from drawing firm conclusions about the translated questionnaire.

## Introduction

Patients’ perceptions are important components of any health technology assessment when developing and introducing technological devices for self-management. Scientific and robust methods are necessary in the evaluation of the technology, including the use of a framework such as the Model of Assessment of Telemedicine [1,2].

In previous research, both qualitative and quantitative research methods and log data from self-monitoring have been used in the evaluation of acceptability. Many published studies use questionnaires [3,4], which are often self-constructed and not validated [4], making the comparison of results across studies difficult. Further, many of these studies are small, with few participants, and have methodological limitations [4]. In particular, limitations related to the development phase and psychometric evaluation of questionnaires measuring patient satisfaction are present, with evaluations lacking data on factor structures, reliability, and validity [5].

There is no consensus related to the definition of the acceptability in mobile health (mHealth) research, although a long list of definitions exists, combining technology and health [6] with users’ perspectives [7]. Previous research has defined users’ perspectives within telemedicine as “issues related to the perception of the patient or the relatives of the telemedicine application including the patients’ and relatives’ acceptance of the technology” [1]. However, we have not been able to find the user perspective defined in terms of mHealth. The *acceptability* of digital solutions in health care is often used synonymously with the concept of satisfaction [7]. In the development of the acceptability questionnaire Service User Technology Acceptability Questionnaire (SUTAQ), Hirani et al aimed to investigate the concept of technology acceptance in more detail [8].

The aim of this study was to assess the validity of the translated Norwegian version of the SUTAQ acceptability questionnaire. This was tested on participants who used an mHealth tool, namely, a digital diabetes diary app running on a mobile phone and a blood glucose meter transferring blood glucose measurements to the app by Bluetooth in the intervention groups of a randomized controlled trial (RCT).

## Methods

### European Union Project

The European Union (EU) project, REgioNs of Europe WorkINg toGether for HEALTH (RENEWING HEALTH), was a research collaboration between 9 regions in Europe working with designing and implementing telemedicine services. The data used in this paper were drawn from the Norwegian study that was a part of this EU project. The acceptability of the equipment was measured at the one-year follow-up in an RCT (NCT01315756).

### Participants and Setting

Persons with type 2 diabetes were randomized to 3 groups. The 2 intervention groups received a diabetes diary app that they had for 1 year, and one of the groups also received health counseling for the first 4 months. In addition, the study had a control group. The participants lived at home and were recruited from primary health care. Of the 101 participants who were randomized to the 2 intervention groups, 74.3% (75/101) completed the SUTAQ questionnaire. Other results from the RCT are reported in detail elsewhere [9-12].

### Service User Technology Acceptability Questionnaire

The SUTAQ was developed for the Whole Systems Demonstrator (WSD) study in the United Kingdom, to measure acceptability and identify the characteristics of persons who were likely to reject technological health services (see Multimedia Appendix 1) [8]. The questionnaire has 22 items, measured on a Likert-scale from 1 to 6, reflecting more or less agreement with the item statements, respectively. The questionnaire has 5 subscales, where each contains between 3 and 9 items. The subscale containing 9 items was further divided into 2. The original items and the subscales are presented later in the paper. The original questionnaire was found to be reliable and valid [8].

As the partners in the RENEWING HEALTH study in 2011 had decided to include answers to SUTAQ in the minimum common dataset, the questionnaire was also used in the Norwegian trial, even though our data collection had already started. The questionnaire was not available in Norwegian when this study started. However, the translation process followed the procedure recommended by the European Organization for Research and Treatment of Cancer Quality of Life Group [13] and the published guidelines for cognitive interviews [14,15]. Two professional translators translated the SUTAQ questionnaire from English to Norwegian. The Norwegian research team considered the discrepancy between the 2 translated versions and the English version. We achieved equivalence with regard to aspects such as the meaning of words, expressions, concepts, and cultural context. A cultural adaptation of the questionnaire had to be done only for a few statements.

A native English speaker, a bilingual person, without any initial knowledge of the SUTAQ, backward translated the final Norwegian version. The research team, also with a good command of English, compared the backward translation with the original questionnaire, and no further changes were made.

Finally, we conducted cognitive interviews with 10 random participants who had answered the SUTAQ questionnaire. According to these interviews, the items were understandable to the participants, although some found the language somewhat cumbersome, leading us to make a few adjustments.

The report from the translation process can be obtained from the last author (LR).

### Statistical Analysis

The sample was described using descriptive statistics. To assess the construct validity of the present domains in the SUTAQ questionnaire from the WSD study, we conducted a confirmatory principal component factor analysis on the 22 items, with Varimax rotation and with a fixed number of 5 factors in accordance with the WSD study [8]. To assess the internal consistency of each domain or extracted factor and for the entire questionnaire, we calculated Cronbach alphas. All analyses were performed using IBM SPSS Statistics v23 (IBM Corp, Armonk, NY, USA).

## Results

### Sample Characteristics

In total, we analyzed data from 75 participants, of whom 56% (42/75) were female. The age range was 35-80 years, with a median age of 59 years, and 49% (37/75) had ≥12 years of education. There were no differences between the 2 intervention groups for the SUTAQ findings. We found no differences in the baseline measures between the 75 participants included in the analyses and the 26 who dropped out during the study. More details concerning demographic and clinical results from the study sample are published elsewhere [16].

**Figure 1:**
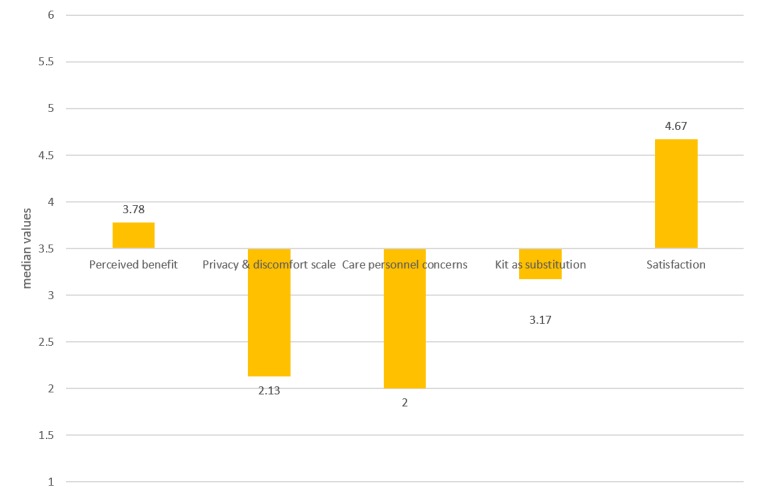
Median reported scores of the Service User Technology Acceptability Questionnaire domains.

The median values for the original SUTAQ domains are presented in Figure 1, indicating that the participants accepted the equipment to a high degree within the 3 areas of *privacy and discomfort*, *care personnel concerns*, and *satisfaction*. This implies a high degree of acceptability regarding beliefs about the security of the monitored data, the impact of the equipment on the user, beliefs of the continuity and skills of the health care personnel facilitating the equipment, and acceptance and satisfaction with the equipment and the given service. The median value between 1 and 6 constitutes the middle value in the figure. The two categories, *privacy and discomfort* and *care personnel concerns* are based on items with negative statements, where high values reflect a high degree of agreement with the negative statements in these two categories, which means that low values represent a positive score. The remaining factors consist of positive statements. High values reflect a high degree of agreement. The participants reported being slightly more than medium positive concerning whether the equipment could improve their care or increase their access to health care within the domain *perceived benefit*. Results from the domain *kit as substitution* indicated that the participants were most critical about the statements concerning this digital solution replacing usual care.

### Factorial Reliability and Validity

The measurement properties of the SUTAQ are presented in Table 1. Overall, the amount of missing data was minimal, no more than 8% for all items. The floor effect was small; only 4 items were far above 15%, considered to be problematic [17]. However, the number of items with ceiling effects was higher, with only about half of the items below the limit of 15%, and for 5 of the items, around 50% (34-40/75) of the participants reached the highest possible score.

The confirmatory factor analysis revealed that only factor 1 and factor 3 were consistent in the original study and this study (Table 2). The first factor, *Perceived benefit*, had 9 items in the original factor structure. Of the items in the Norwegian dataset, 7 loaded >0.400, which was the limit within the factors in the WSD study [8]. In the third domain, *Care personnel concerns*, all 3 items loaded >0.400. The Cronbach alpha coefficient for all 22 items was .851, which demonstrates good internal consistency [18]. Cronbach alpha values for each factor are listed in Table 2.

**Table 1:** Service User Technology Acceptability Questionnaire item descriptors.

Items (range 1-6)	Median	Missing, n (%)	Floor, n (%)	Ceiling, n (%)
The kit I received has saved me time in that I did not have to visit my GP clinic or other health/social care professional as often	4	4 (5)	1 (1)	17 (23)
The kit I received has interfered with my everyday routine	5	3 (4)	2 (3)	35 (47)
The kit I received has increased my access to care (health and/or social care professionals)	5	4 (5)	4 (6)	24 (33)
The kit I received has helped me to improve my health	3	3 (4)	7 (10)	8 (11)
The kit I received has invaded my privacy	5	4 (5)	2 (3)	23 (32)
The kit has been explained to me sufficiently	2	3 (4)	26 (35)	2 (3)
The kit can be trusted to work appropriately	2	3 (4)	17 (23)	10 (14)
The kit has made me feel uncomfortable, eg, physically or emotionally	6	3 (4)	2 (3)	40 (54)
I am concerned about the level of expertise of the individuals who monitor my status via the kit	6	5 (7)	0 (0)	40 (56)
The kit has allowed me to be less concerned about my health and/or social care	3.5	5 (7)	4 (6)	9 (13)
The kit has made me more actively involved in my health	3	5 (7)	7 (10)	8 (11)
The kit makes me worried about the confidentiality of the private information being exchanged through it	5	5 (7)	5 (7)	34 (47)
The kit allows the people looking after me, to better monitor me and my condition	3	5 (7)	11 (15)	8 (11)
I am satisfied with the kit I received	2	4 (5)	11 (15)	10 (14)
The kit can be/should be recommended to people in a similar condition to mine	2	5 (7)	18 (25)	7 (10)
The kit can be a replacement for my regular health or social care	4	5 (7)	5 (7)	17 (24)
The kit can certainly be a good addition to my regular health or social care	2	5 (7)	20 (28)	6 (8)
The kit is not as suitable as regular face to face consultations with the people looking after me	3	4 (5)	13 (18)	4 (6)
The kit has made it easier to get in touch with health and social care professionals	4	5 (7)	4 (6)	19 (26)
The kit interferes with the continuity of the care I receive (ie, I do not see the same care professional each time)	5	6 (8)	1 (1)	34 (48)
I am concerned that the person who monitors my status, through the kit, does not know my personal health/social care history	5	6 (8)	3 (4)	22 (31)
The kit has allowed me to be less concerned about my health status	3	5 (7)	6 (8)	11 (15)

**Table 2:** Confirmatory factor analysis showing Cronbach alpha values.

Item	Factor 1: perceived benefit	Factor 2: privacy and discomfort	Factor 3: care personnel concerns	Factor 4: satisfaction	Factor 5: kit as substitution
The kit can be/should be recommended to people in a similar condition to mine	*.880* ^a,b^	.146	.060	−.077	.079
The kit can certainly be a good addition to my regular health or social care	*.821* ^a,b^	.065	−.022	−.101	.220
I am satisfied with the kit I received	*.815* ^a^	.257	.028	−.121^b^	.093
The kit has made me more actively involved in my health	*.779* ^a,b^	.202	−.026	.253	−.098
The kit I received has helped me to improve my health	*.709* ^a,b^	.276	−.132	.181	−.098
The kit has allowed me to be less concerned about my health status	*.693* ^a^	.125	.050	−.168	−.005^b^
The kit has allowed me to be less concerned about my health and/or social care	*.676* ^a,b^	.201	.057	.028	−.194
The kit can be trusted to work appropriately	*.682* ^a^	.103	−.165	.066^b^	−.263
The kit allows the people looking after me to better monitor me and my condition	*.650* ^a,b^	.292	.043	−.395	.072
The kit has been explained to me sufficiently	*.505* ^a^	−.022	−.084	−.394^b^	.443
The kit I received has saved me time in that I did not have to visit my GP clinic or other health/social care professional as often	.291^b^	*.751* ^a^	−.057	.006	.100
The kit has made it easier to get in touch with health and social care professionals	.402^b^	*.721* ^a^	−.004	.134	−.067
The kit I received has increased my access to care (health and/or social care professionals)	.246^b^	*.668* ^a^	.205	.042	−.131
The kit can be a replacement for my regular health or social care	.411	*.612* ^a^	.169	−.243	−.117^b^
I am concerned that the person who monitors my status, through the kit, does not know my personal health/social care history	.119	−.048	*.824* ^a,b^	.204	.234
The kit makes me worried about the confidentiality of the private information being exchanged through it	−.070	.130^b^	*.791* ^a^	.095	.116
I am concerned about the level of expertise of the individuals who monitor my status via the kit	.038	−.040	*.738* ^a,b^	.210	−.341
The kit interferes with the continuity of the care I receive (ie, I do not see the same care professional each time)	−.199	.383	*.656* ^a,b^	.122	.318
The kit I received has invaded my privacy	.051	−.069^b^	.281	*.774* ^a^	.065
The kit I received has interfered with my everyday routine	−.118	.187^b^	.336	*.606* ^a^	.159
The kit is not as suitable as regular face to face consultations with the people looking after me	−.154	.287	−.223	−.138	*−.722* ^a,b^
The kit has made me feel uncomfortable, eg, physically or emotionally	−.031	.420^b^	.243	.359	*.536* ^a^
Cronbach alpha	.892	.721	.701	.766	.295
Explained variance, %	31.3	16.4	8.2	5.5	5.1

^a^Italicized values indicate loading in the present Norwegian data.

^b^Original loading in the Whole Systems Demonstrator study.

## Discussion

### Principal Findings

The Norwegian version of SUTAQ revealed good internal consistency, with a Cronbach alpha of .851. However, the original five-factor solution was not confirmed. On the contrary, our results indicated that a one-factor solution, or at most a three-factor solution, was sufficient, as the explained variance increased by <6% when adding more factors (Table 2). Moreover, only 2 items were loaded on each of the last factors (factors 4 and 5), indicating that they were superfluous. In addition, we found that the SUTAQ questionnaire had some items with a floor effect and even more items with ceiling effects.

### Limitations

One limitation of this study was the low number of participants, as over 250 or at least 10 participants per item is recommended to enable precise conclusions from factor analysis [19]. Further, a factor loading above 0.7 per item is preferred according to Kaiser’s criteria [20]. Thus, the small sample size might be one of the possible explanations for the lack of confirmation of all factors. Exploratory factor analysis would have been a suitable statistical method to explore the potential of the questionnaire in our Norwegian setting, although demanding a larger number of participants.

Differences in study contexts, health issues, and equipment could also contribute to the lack of common factors in the original study and this study. In the WSD study, interventions were given to patients with long-term conditions, not only diabetes but also chronic obstructive pulmonary disease, heart failure, and social needs [21]. Further, a far broader range of equipment was used in the WSD study: both telehealth and telecare. In this study, only persons with type 2 diabetes used the self-management app, and no telemonitoring was involved. Outdated equipment was also a problem in the Norwegian study because of a long inclusion process [10].

Our data were slightly skewed (Table 1), and to our knowledge, there are no references to an acceptable level of floor and ceiling effects in similar technological studies. Quality criteria available in the literature suggest that floor or ceiling effects over 15% will reduce the reliability of the item in health status questionnaires. In addition, such an item cannot distinguish between the groups of responders scoring at either end of the scale [17]. Only 6 of the 22 items had an acceptable level (≤15%) of both floor and ceiling effects. Other SUTAQ studies [8,22] did not report on the floor and ceiling effects of each item but did present histograms and means for the domains. It seems that the data on the domains *Satisfaction* and *Privacy and discomfort* were skewed in those studies [8,22]. Hirani et al [8] explained the skewedness of items as being linked to the dropout rate from their study, as persons dropping out could have scored somewhat different from the remaining participants, possibly leading to bias and reduced generalizability. The responders were expected to be more satisfied than nonresponders; this explanation could also be relevant for our Norwegian study. However, even if the remaining participants were more satisfied, the questionnaire did not capture details of their satisfaction.

Using an unvalidated questionnaire is a limitation as described by Streiner [18]. This refers both to the development of the questionnaire and to the generalizability of the translated version, which may lack equivalence with the original questionnaire. Being part of a large EU study, we agreed upon the selection of common questionnaires. Before our one-year follow-up, the partners decided to introduce the SUTAQ. At that time, we translated the instrument according to standardized procedures for translation [13]. This gave us knowledge about the participants’ conceptual and semantic understanding of the items. If we had the opportunity to perform a questionnaire validation of the SUTAQ ahead of the study, this would have improved reflections about its validity. Another aspect is that SUTAQ was developed for the WSD study evaluating different technologies and measuring the acceptability of telehealth and telecare interventions, with a closer follow-up from health care personnel than that in the Norwegian self-management study. The differences in the content of the interventions between the original [8] and this mHealth study could have affected the validation analysis, as the SUTAQ might be more suitable for a different type of intervention than the one implemented in this study. Finally, even though we carefully followed the translation procedures, we cannot rule out the risk that the translation from English to Norwegian could have changed the understanding of the initial meaning of the statements in SUTAQ.

Originally, we aimed to perform a test-retest analysis to measure reliability, which would require data on 40-50 participants. Unfortunately, we did not reach the sufficient number of participants because of financial and logistical difficulties. We measured acceptability at the last point of follow-up in the study, making it difficult to collect additional retest questionnaires. Given that we had only 12 retest responders, we realized that we did not have enough statistical power to perform a meaningful test-retest analysis.

### Implications for Future Research and Clinical Practice

In the diverse reality of technology and health, it is challenging to measure patient perception. Nevertheless, we are still in need of a questionnaire that measures the acceptability of digital interventions, given the current development and implementation of many new apps and Web solutions in health care. Health technology assessment as a systematic evaluation contributes to the evaluation of various impacts of health technology [23], so there is a need for validated measurements of the acceptability of the technology among users. The SUTAQ measures several such relevant aspects, such as the impact on relations to health care personnel, privacy, etc. A relatively small sample size has restrained us from drawing any firm conclusions. SUTAQ should be validated using a larger sample and possibly a modified version developed for use in the Norwegian setting.

## Appendix

Multimedia Appendix 1 The Service User Technology Acceptability Questionnaire (original version; published with permission from Shashi Hirani).

## Abbreviations

EU: European Union
mHealth: mobile health
RCT: randomized controlled trial
RENEWING HEALTH: REgioNs of Europe WorkINg toGether for HEALTH
SUTAQ: Service User Technology Acceptability Questionnaire
WSD: Whole Systems Demonstrator
