# Supplementary material for: The Service User Technology Acceptability Questionnaire: Psychometric Evaluation of the Norwegian Version
Source: JMIR Hum Factors. 2018 Dec 21;5(4):e10255. doi: 10.2196/10255 (PMC6324518; doi:10.2196/10255)
Supplement: Multimedia Appendix 1 [file humanfactors_v5i4e10255_app1.pdf]

Instructions: Below is a list of statements referring to the kit (TeleHealth and/or TeleCare equipment) you have received to support your care. Please indicate the degree to which you agree with each statement by **TICKING** the corresponding box.

1. The kit I received has saved me time in that I did not have to visit my GP clinic or other health/social care professional as often.

☐	☐	☐	☐	☐	☐
STRONGLY AGREE	MODERATELY AGREE	MILDLY AGREE	MILDLY DISAGREE	MODERATELY DISAGREE	STRONGLY DISAGREE

2. The kit I received has interfered with my everyday routine.

☐	☐	☐	☐	☐	☐
STRONGLY AGREE	MODERATELY AGREE	MILDLY AGREE	MILDLY DISAGREE	MODERATELY DISAGREE	STRONGLY DISAGREE

3. The kit I received has increased my access to care (health and/or social care professionals).

☐	☐	☐	☐	☐	☐
STRONGLY AGREE	MODERATELY AGREE	MILDLY AGREE	MILDLY DISAGREE	MODERATELY DISAGREE	STRONGLY DISAGREE

4. The kit I received has helped me to improve my health.

☐	☐	☐	☐	☐	☐
STRONGLY AGREE	MODERATELY AGREE	MILDLY AGREE	MILDLY DISAGREE	MODERATELY DISAGREE	STRONGLY DISAGREE

5. The kit I received has invaded my privacy.

☐	☐	☐	☐	☐	☐
STRONGLY AGREE	MODERATELY AGREE	MILDLY AGREE	MILDLY DISAGREE	MODERATELY DISAGREE	STRONGLY DISAGREE

6. The kit has been explained to me sufficiently.

☐	☐	☐	☐	☐	☐
STRONGLY AGREE	MODERATELY AGREE	MILDLY AGREE	MILDLY DISAGREE	MODERATELY DISAGREE	STRONGLY DISAGREE

7. The kit can be trusted to work appropriately.

☐	☐	☐	☐	☐	☐
STRONGLY AGREE	MODERATELY AGREE	MILDLY AGREE	MILDLY DISAGREE	MODERATELY DISAGREE	STRONGLY DISAGREE

8. The kit has made me feel uncomfortable, e.g. physically or emotionally.

☐	☐	☐	☐	☐	☐
STRONGLY AGREE	MODERATELY AGREE	MILDLY AGREE	MILDLY DISAGREE	MODERATELY DISAGREE	STRONGLY DISAGREE

9. I am concerned about the level of expertise of the individuals who monitor my status via the kit.

☐	☐	☐	☐	☐	☐
STRONGLY AGREE	MODERATELY AGREE	MILDLY AGREE	MILDLY DISAGREE	MODERATELY DISAGREE	STRONGLY DISAGREE

10. The kit has allowed me to be less concerned about my health and/or social care.

☐	☐	☐	☐	☐	☐
STRONGLY AGREE	MODERATELY AGREE	MILDLY AGREE	MILDLY DISAGREE	MODERATELY DISAGREE	STRONGLY DISAGREE

11. The kit has made me more actively involved in my health.

☐	☐	☐	☐	☐	☐
STRONGLY AGREE	MODERATELY AGREE	MILDLY AGREE	MILDLY DISAGREE	MODERATELY DISAGREE	STRONGLY DISAGREE

12. The kit makes me worried about the confidentiality of the private information being exchanged through it.

☐	☐	☐	☐	☐	☐
STRONGLY AGREE	MODERATELY AGREE	MILDLY AGREE	MILDLY DISAGREE	MODERATELY DISAGREE	STRONGLY DISAGREE

13. The kit allows the people looking after me, to better monitor me and my condition.

☐	☐	☐	☐	☐	☐
STRONGLY AGREE	MODERATELY AGREE	MILDLY AGREE	MILDLY DISAGREE	MODERATELY DISAGREE	STRONGLY DISAGREE

14. I am satisfied with the kit I received.

☐	☐	☐	☐	☐	☐
STRONGLY AGREE	MODERATELY AGREE	MILDLY AGREE	MILDLY DISAGREE	MODERATELY DISAGREE	STRONGLY DISAGREE

15. The kit can be/should be recommended to people in a similar condition to mine.

☐	☐	☐	☐	☐	☐
STRONGLY AGREE	MODERATELY AGREE	MILDLY AGREE	MILDLY DISAGREE	MODERATELY DISAGREE	STRONGLY DISAGREE

16. The kit can be a replacement for my regular health or social care.

☐	☐	☐	☐	☐	☐
STRONGLY AGREE	MODERATELY AGREE	MILDLY AGREE	MILDLY DISAGREE	MODERATELY DISAGREE	STRONGLY DISAGREE

17. The kit can certainly be a good addition to my regular health or social care.

☐	☐	☐	☐	☐	☐
STRONGLY AGREE	MODERATELY AGREE	MILDLY AGREE	MILDLY DISAGREE	MODERATELY DISAGREE	STRONGLY DISAGREE

18. The kit is not as suitable as regular face to face consultations with the people looking after me.

☐	☐	☐	☐	☐	☐
STRONGLY AGREE	MODERATELY AGREE	MILDLY AGREE	MILDLY DISAGREE	MODERATELY DISAGREE	STRONGLY DISAGREE

19. The kit has made it easier to get in touch with health and social care professionals.

☐	☐	☐	☐	☐	☐
STRONGLY AGREE	MODERATELY AGREE	MILDLY AGREE	MILDLY DISAGREE	MODERATELY DISAGREE	STRONGLY DISAGREE

20. The kit interferes with the continuity of the care I receive (i.e. I do not see the same care professional each time).

☐	☐	☐	☐	☐	☐
STRONGLY AGREE	MODERATELY AGREE	MILDLY AGREE	MILDLY DISAGREE	MODERATELY DISAGREE	STRONGLY DISAGREE

21. I am concerned that the person who monitors my status, through the kit, does not know my personal health/social care history.

☐	☐	☐	☐	☐	☐
STRONGLY AGREE	MODERATELY AGREE	MILDLY AGREE	MILDLY DISAGREE	MODERATELY DISAGREE	STRONGLY DISAGREE

22. The kit has allowed me to be less concerned about my health status.

☐	☐	☐	☐	☐	☐
STRONGLY AGREE	MODERATELY AGREE	MILDLY AGREE	MILDLY DISAGREE	MODERATELY DISAGREE	STRONGLY DISAGREE

Thank you for your responses to this questionnaire, please check that you have answered all items. Your responses will be kept confidential
